# The metabolic footprint of Vero E6 cells highlights the key metabolic routes associated with SARS-CoV-2 infection and response to drug combinations

**DOI:** 10.1038/s41598-024-57726-3

**Published:** 2024-04-04

**Authors:** Riccardo Melis, Angela Braca, Daniela Pagnozzi, Roberto Anedda

**Affiliations:** Porto Conte Ricerche s.r.l., S.P. 55 Porto Conte-Capo Caccia, Km 8.400 Loc. Tramariglio, Alghero, SS Italy

**Keywords:** SARS-CoV-2, Remdesivir, Azithromycin, NMR footprint, Vero E6 cells, Metabolomics, Biomarkers

## Abstract

SARS-CoV-2 burdens healthcare systems worldwide, yet specific drug-based treatments are still unavailable. Understanding the effects of SARS-CoV-2 on host molecular pathways is critical for providing full descriptions and optimizing therapeutic targets. The present study used Nuclear Magnetic Resonance-based metabolic footprinting to characterize the secreted cellular metabolite levels (exometabolomes) of Vero E6 cells in response to SARS-CoV-2 infection and to two candidate drugs (Remdesivir, RDV, and Azithromycin, AZI), either alone or in combination. SARS-CoV-2 infection appears to force VE6 cells to have increased glucose concentrations from extra-cellular medium and altered energetic metabolism. RDV and AZI, either alone or in combination, can modify the glycolic-gluconeogenesis pathway in the host cell, thus impairing the mitochondrial oxidative damage caused by the SARS-CoV-2 in the primary phase. RDV treatment appears to be associated with a metabolic shift toward the TCA cycle. Our findings reveal a metabolic reprogramming produced by studied pharmacological treatments that protects host cells against virus-induced metabolic damage, with an emphasis on the glycolytic-gluconeogenetic pathway. These findings may help researchers better understand the relevant biological mechanisms involved in viral infection, as well as the creation of mechanistic hypotheses for such candidate drugs, thereby opening up new possibilities for SARS-CoV-2 pharmacological therapy.

## Introduction

The recent Coronavirus 2019 (COVID-19) pandemic produced by SARS coronavirus 2 (SARS-CoV-2) has become a huge threat to global health^1^. Despite major efforts to swiftly develop vaccine therapies to manage pandemic phenomena^2^, unexpected outbreaks and the increasing prevalence of virus mutation resistance are expected. Meanwhile, several therapy techniques are being attempted to enhance the benefit/risk ratio of COVID-19 patients^3^. Pharmaceutical firms are constantly attempting to identify novel compounds to combat COVID-19, as well as assessing the benefits of repurposing existing authorized medications. Indeed, certain pre-existing drugs have demonstrated strong therapeutic effectiveness against SARS-CoV-2, and their repurposing can be advantageous due to lower costs and a faster regulatory clearance procedure^4^.

Remdesivir (RDV), the first drug that received approval from the Food and Drug Administration (FDA) for the treatment of COVID-19, is a nucleotide prodrug metabolized into an adenosine triphosphate analog (GS-441544, Nuc). RDV acts as an RNA polymerase inhibitor, incorporating into nascent viral RNA chains and leading to their premature termination^5^. RDV has previously evidenced a broad-spectrum activity against several other viruses and, more recently, an interesting pharmacological activity against SARS-CoV-2^3,6^.

Another type of prospective supplementary pharmacological therapy is the use of non-directed antiviral medicines, which work against the inflammatory and innate immune responses generated by SARS-CoV-2 infection^7^. Azithromycin (AZI), an antibiotic belonging to the macrolide family commonly adopted against bacterial diseases, has been recently proposed—alone or in combination with other drugs—as a potential therapeutic agent for the treatment of SARS-CoV-2-induced pneumonia^8^.

Among the numerous ways being researched are synergistic drug combinations, which may provide improved coverage in poly-viral prophylaxis as well as therapy of caused pathological conditions. Furthermore, combining potential medications against SARS-CoV-2 is consistent with the treatment of other viral diseases^9^. In addition to prompting research into the effective clinical usefulness of such drug-based treatments, there is an urgent need to expand our understanding of such drug-induced metabolic abnormalities. Indeed, the molecular mechanisms of both RDV and AZI are not entirely understood, particularly their caused metabolic perturbations.

Metabolomics is one of the most powerful and highly effective methodological tools to investigate the interaction between genetic background, exogenous and endogenous factors, and their effect on human health. Pharmacometabolomics, in particular, is concerned with the characterization of metabolic profiles or *metabotype* alterations generated by drug treatments, hence giving a direct reflection of cells/tissues' metabolic condition and aiding a better understanding of linked biological processes^10^. Although intrinsically not very sensitive compared to other analytical platforms such as mass spectrometry, Proton Nuclear Magnetic Resonance (^1^H NMR)-based metabolomics has the indubitable advantage of being able to recognize metabolites, measure their relative or absolute abundances, and identify perturbations in the biochemical pathways in a highly reproducible and fast manner, with a minimal sample handling and short experimental time^11^. In particular, as the composition of the extracellular medium is known to be highly correlated with alterations in intracellular metabolism^12,13^, the NMR-based metabolic characterization of the extracellular metabolites consumed from or excreted to the media (*metabolic footprint*) has been demonstrated to be extremely useful to identify molecular biomarkers as prognostic or diagnostic tools^14–16^.

In agreement with the aforementioned shreds of evidence, the goal of this study was to look at the changes in the levels of metabolites secreted or absorbed by Vero E6 cells (*exometabolomes*) in response to viral SARS-CoV-2 infection and as a result of pre-treatment with RDV and AZI, either alone or in combination.

The study's goal is to confirm the metabolic impact of SARS-CoV-2 infection adopting a scientific approach that is closely consistent with previous in-vitro and in-vivo research. Furthermore, our work aims to make some important contributions to understanding how RDV and AZI (alone or in combination) affect the release/absorption of small metabolites from host cells, and how such candidate drugs may prevent or reduce SARS-CoV-2-virus-induced metabolic impairments through extensive host cell metabolic reprogramming.

## Results

### ^1^H NMR characterization of the VE6 cell exometabolome

The ^1^H NMR analysis allowed us to unambiguously identify 22 small extra-cellular metabolites, such as organic acids (mostly lactic and acetic acid), carbohydrates (D-glucose), amino acids (AA), osmolites as well as nucleotides (Supplementary Table S1 and Supplementary Figure S1) and to perform a relative quantification on them.

### NMR footprint following SARS-CoV-2 infection and drug treatments

**Figure 1:**
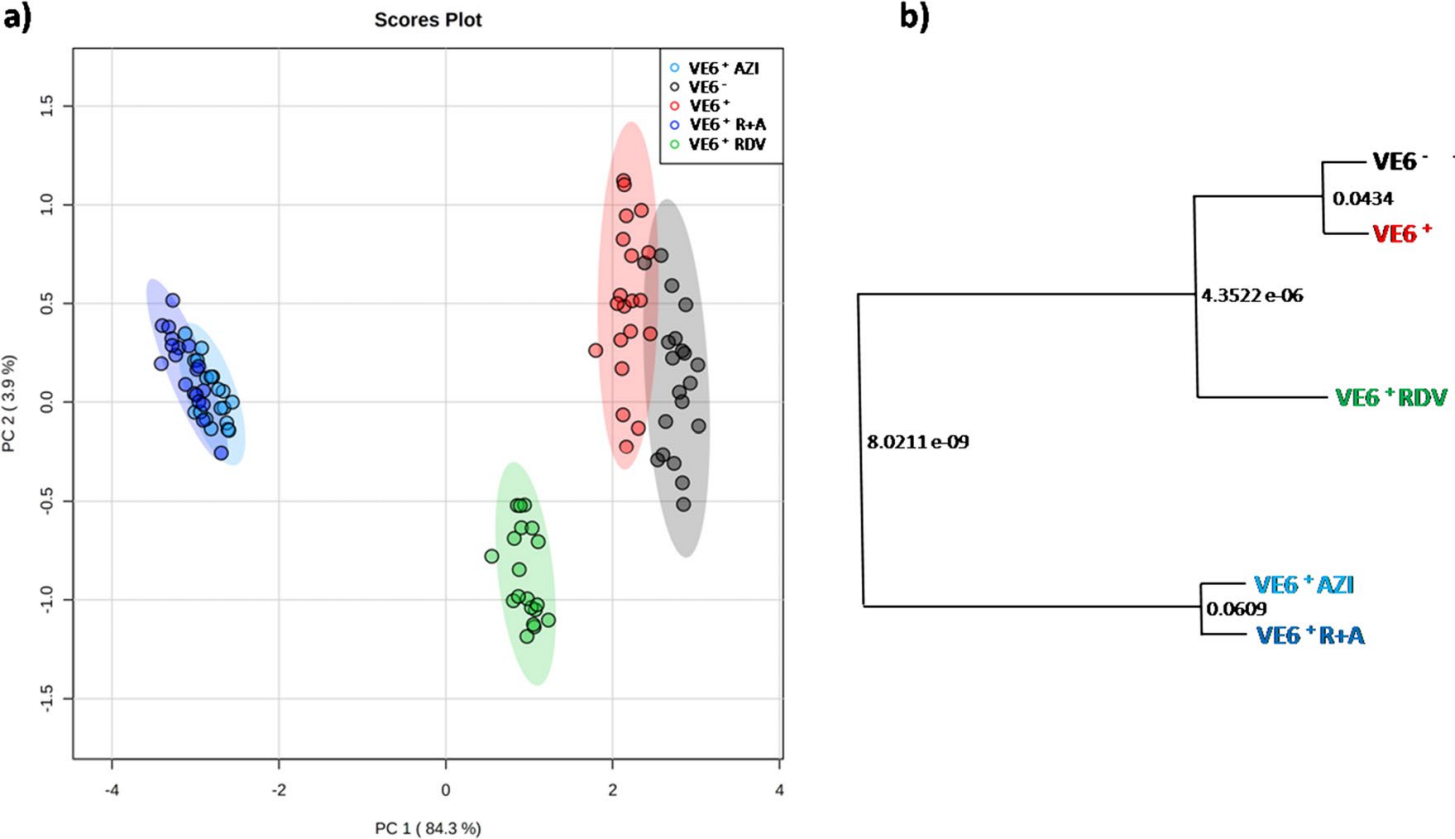
Unsupervised MVDA of VE6 exometabolomes: (**a**) PCA score plot, where each point confined in Hotelling’s T^2^ ellipsoids corresponds to NMR data from each cell supernatant replicate. The grouping association to treatments is outlined by a specific color code: VE6 (gray dots); VE6^+^ (red dots); VE6^+^RDV (green dots); VE6^+^AZI (cyan dots); VE6^+^R + A (blue dots). Percentages explained by the first (PC 1) and second (PC 2) components were also reported. (**b**) PCAToTree2 dendrogram corresponding to the computed PCA score plots using Mahalanobis distances, with p-values for the null hypothesis (p < 0.05) at each branch.

Multivariate data analysis (MVDA, Fig. 1) was used to examine the ^1^H NMR integrals associated with the identified compounds in the VE6 cells exometabolomes (Supplementary Table S1).

A first exploratory PCA scores plot reveals the cluster separations of VE6 exometabolomes according to both viral infection and drug treatments (Fig. 1a). Notably, the scores associated with the VE6^+^RDV and VE6^+^AZI/VE6^+^R + A groups were well separated from those related to the untreated groups. Contrarily, the distinction between V6^−^ and VE6^+^ was less apparent, with even greater overlap observed between the VE6^+^AZI and VE6^+^ R + A pair (Fig. 1a). The high value of the variability explained by the first component (PC1 > 84%) indicates an excellent model validation.

The adoption of the PCA2tree approach allows for a more in-depth statistical examination of the observed clustering. As highlighted in Fig. 1b, both single-drug treatments and the synergistic approach appear to have a stronger effect on the VE6 extra-cellular metabolite levels compared to SARS-CoV-2 infection only, being the difference between untreated positive and negative cell lines less significant (p < 0.05) than treated cells VE6^+^RDV (p > 0.06) [or VE6^+^AZI and VE6^+^ R + A (p << 0.001)]. No significant branch separation was instead computed between VE6^+^AZI and VE6^+^ R + A groups (p > 0.05).

Supervised MVDA (PLS-DA) was then used to compare in more detail the exometabolomes according to the following comparisons: (a) VE6^+^ vs. VE6^−^; (b) VE6^+^RDV vs. VE6^+^; (c) VE6^+^AZI vs. VE6^+^; and (d) VE6^+^R + A vs. VE6^+^ (Supplementary Figure S2).

In agreement with the above-discussed unsupervised MVDA inspection results, related PLS-DA cross-validation analyses provide excellent prediction parameters (Q2 > 0.8), with the only exception of the comparison (a) (VE6^−^ vs. VE6^+^) that exhibits a lower, though still reliable, predictive ability (Q2 < 0.6) (Supplementary Figure S3).

**Figure 2:**
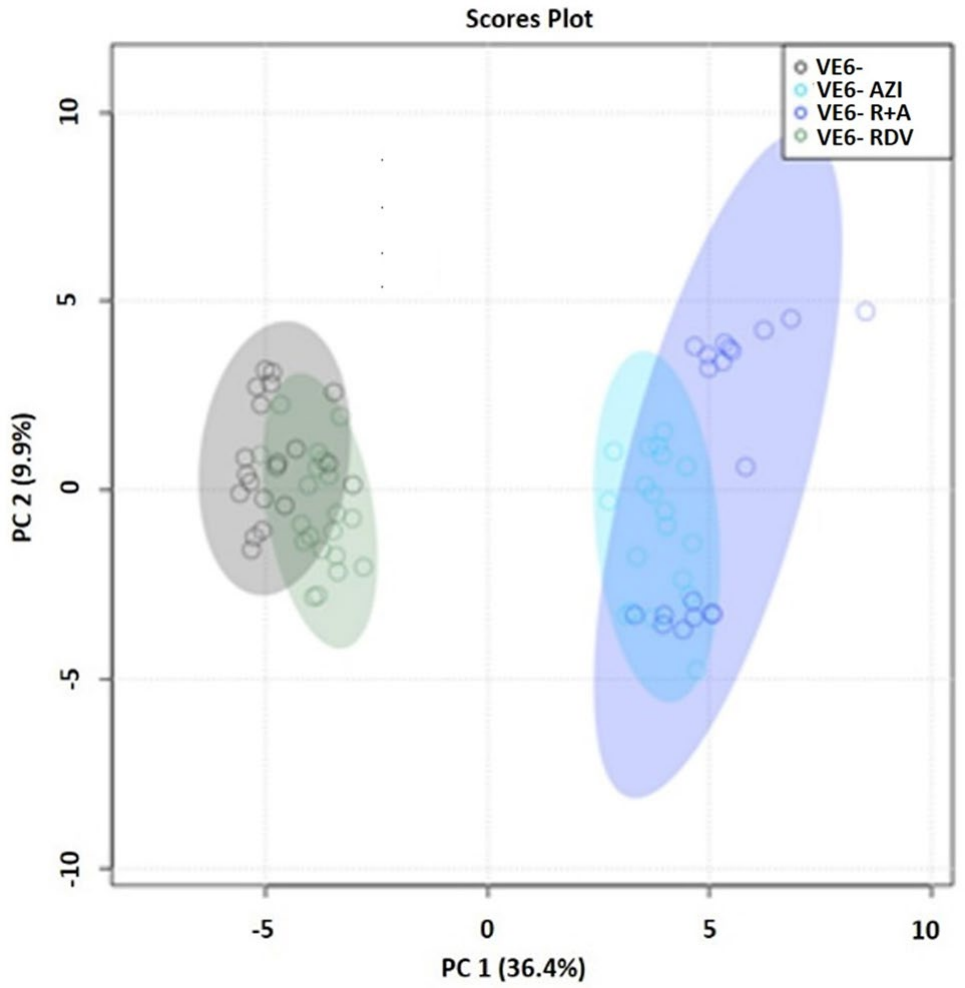
PCA score plot related to exometabolomes of uninfected untreated and treated VE6 cells. The grouping of NMR profiles of supernatants according to treatments is depicted using a designated color scheme: uninfected (VE6-) represented by gray dots; VE6-RDV treatment indicated by dark green dots; VE6-AZI treatment by cyan dots; and VE6-R + A treatment by blue dots. Additionally, the percentages of variance explained by the first (PC 1) and second (PC 2) principal components are provided.

The additional MVDA of the NMR omics response determined by the administration of antivirals on uninfected cell lines revealed a partially different multivariate pattern compared to that extrapolated from infected lines. From the corresponding PCA score plot (Fig. 2), clear differentiation of clusters related to uninfected lines treated with AZI (VE6^−^AZI) and in synergy (VE6^−^R + A) is observed, which again appear interpenetrated and well separated from the non-infected control (VE6^−^). However, the clusters related to uninfected (VE6^−^) lines and those of uninfected lines treated with RDV (VE6^−^RDV) were significantly overlapping, unlike what was previously observed in the previously shown pattern extrapolated from infected lines (Fig. 1).

### Metabolic perturbations associated with SARS-CoV-2 infection and therapy

**Figure 3:**
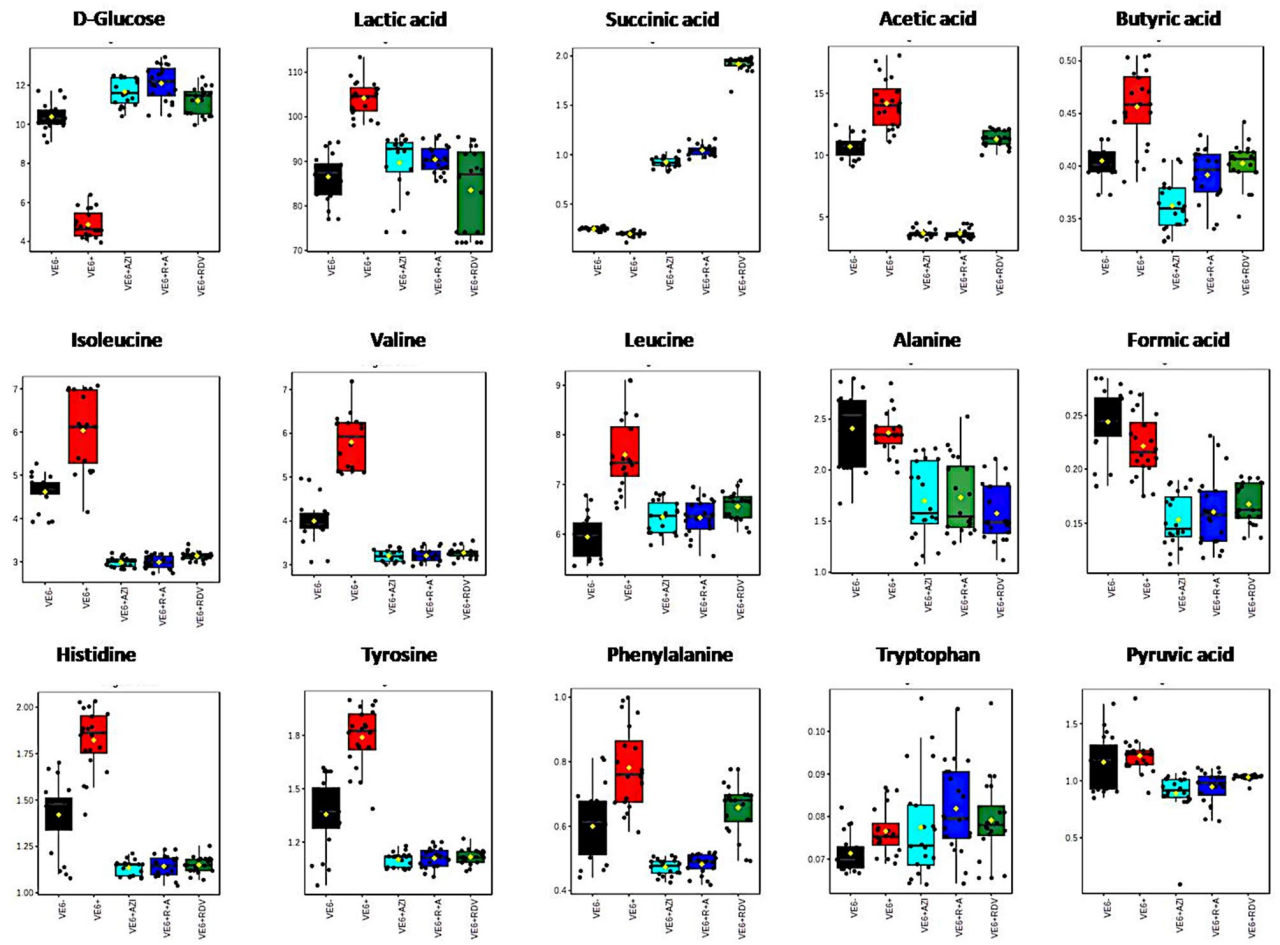
Box plots reporting some of the most significant changes in the metabolites detected in the extra-cellular medium of VE6^−^ (black), VE6^+^ (red), VE6^+^ RDV (green), VE6^+^ AZI (green) and VE6^+^ R + A (blue) cell lines.

The importance feature analysis based on univariate statistical approaches allowed us to gain information on the metabolites responsible for all group inter-comparisons. Representative box plots depicted in Fig. 3 denote the most significantly altered metabolites associated with each examined treatment.

Overall, SARS-CoV-2 infection led to a notable reduction in glucose levels coupled with a significant elevation in organic acids (such as acetic and lactic acids) as well as various branched-chain (leucine, isoleucine, valine, and alanine) and aromatic amino acids (histidine, phenylalanine, and tyrosine). Conversely, administration of the candidate drugs alone or in combination resulted in significant alterations (either increases or decreases) in the levels of several of these metabolites compared to both untreated VE6- and VE6 + cells, underscoring the potent metabolic effects of the drug treatments tested.

While it is important to highlight a pertinent feature, such as the heightened secretion of succinic acid observed in the VE6 + RDV group, which stands out notably in comparison to other treatments (see Fig. 3), the variations in other metabolites between VE6 + RDV and VE6 + AZI, as well as in comparison to VE6 + R + A, were comparatively less pronounced, as extensively elaborated in the Supplementary Materials (see Figure S4 and Zenodo (https://zenodo.org/records/10158609).

**Figure 4:**
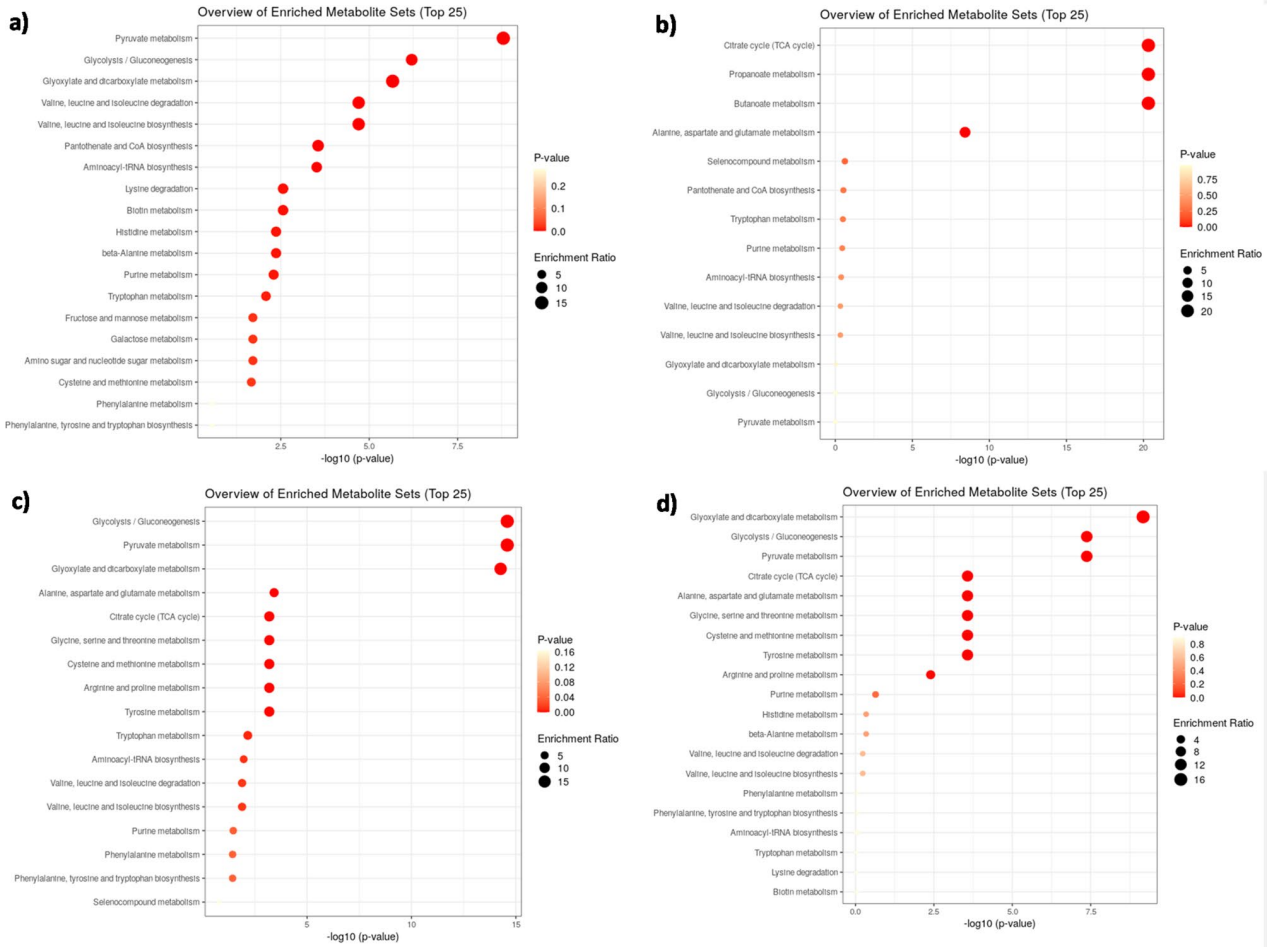
MSEA performed on NMR data (integrals, a.u.) following the comparisons between: (**a**) VE6^+^ vs. VE6^−^; (**b**) VE6^+^RDV vs. VE6^+^; (**c**) VE6^+^AZI vs. VE6^+^; and (**d**) VE6^+^R + A vs. VE6^+^. For each diagram, the y-axis represents the pathway impact value indicated by the Enrichment Ratio (ER), and the x-axis the statistical significance indicated by the logarithmic form of p-values. The gradient color and the radius of the displayed nodes are respectively related to their p-values and to their pathway impact by ER.

To identify the key metabolic perturbations associated with the described extra-cellular metabolic alterations, the same NMR data were used to perform MSEA analysis, which provides a more comprehensive overview of the many individual metabolic changes observed. Following the PLS-DA above-reported comparisons, MSEA produced a list of the top metabolic pathways sorted in order of significance parameters based on either the pathway impact or p-values (Fig. 4).

A first MSEA-based comparison between infected (VE6^+^) and non-infected (VE6^−^) cells is depicted in Fig. 4a. The main perturbed pathways associated with viral infection include the metabolism of pyruvic acid (*Pyruvate metabolism*, ER = 15,431, p-value = 6,25E−09) and the glycolytic-gluconeogenic pathway (*Glycolysis/Gluconeogenesis*, ER = 11,316, p-value = 4,22E−07), followed by the glyoxylate/dicarboxylate compartment (*Glyoxylate and dicarboxylate* metabolism, ER = 14,067, p-value = 1,75E−06). Other, but less significant perturbations, are related to the biosynthesis/degradation of branched-chain AA (*Valine*, *leucine*, *and Isoleucine biosynthesis* (ER = 12,211, p-value = 1,49E−05) and *degradation* (ER = 12,301, p-value = 1,34E−05), aminoacyl-tRNA biosynthesis pathway (*Aminoacyl-tRNA biosynthesis*, ER = 10,887, p-value = 4,86E−04), and to the synthesis of the main precursor (Vitamin B5) of phospholipids and fatty acids (*Pantothenate* and *CoA biosynthesis*, ER = 10,895, p-value = 3,17E−04).

Accounting for the comparison between VE6^+^RDV and VE6^+^ (Fig. 4b), the more significantly enriched pathways are related to the TCA cycle (*citric acid cycle*, ER = 20,228, p-value = 1,31E−21) as well as to the metabolism of short-chain fatty acids (SCFAs) [*Propanoate metabolism *(ER = 20,217, p-value = 2,37E−21) and *butanoate metabolism* (ER = 20,144, p-value = 2,43E−21)] and, to a lesser extent, to the metabolism of the alanine, aspartate and glutamate [*Alanine*, *aspartate* and *glutamate metabolism*, (ER = 6574, p-value = 5,22E−08)]. Interestingly, the main metabolic perturbations associated with SARS-CoV-2 infection highlighted in Fig. 4a (i.e. *Pyruvate metabolism*, *Glycolysis/Gluconeogenesis*, and *Glyoxylate and dicarboxylate metabolism*) appear to be inversely ranked and shifted at the bottom of the list in Fig. 4b as an effect of administration of RDV. The same can be said for other metabolisms, such as that of branched-chain AA biosynthesis and degradation and the above-mentioned Pantothenate and CoA metabolism.

Surprisingly, the treatment with AZI alone (VE6^+^AZI vs. VE6^+^, Fig. 4c) and the combination of AZI and RDV (VE6^+^R + A vs. VE6^+^, Fig. 4d) resulted in very comparable metabolic perturbations on SARS-CoV-2 infected cells, except for the different statistical significance given by p-values. Moreover, as a consequence of the synergic drug treatment in VE6^+^R + A (Fig. 4d), the TCA cycle appears to have much less impact compared to the treatment with RDV alone (Fig. 4b).

## Discussion

The present study delves into the intricate metabolic shifts within the exometabolomes of VE6 cells in response to both SARS-CoV-2 infection and the preemptive application of RDV and AZI candidate therapeutics, either alone or in combination. Leveraging the precision of ^1^H NMR metabolic profiling, we discerned a spectrum of over twenty vital small metabolites, serving as crucial energy intermediates. This comprehensive analysis affords a meticulous panorama of the VE6 cell-secreted metabolomes amid the explored conditions, illuminating key insights into their dynamic responses.

Several of these extra-cellular metabolite levels appeared to be either significantly increased or reduced in response to the different examined conditions, namely viral infection and drug treatments. This provides a clearer understanding of the metabolic impact of the selected drugs and their pharmacological combinations in this cell model and indirectly offers an interesting picture of the activities of the intracellular VE6 enzymes and associated metabolic pathways.

**Figure 5:**
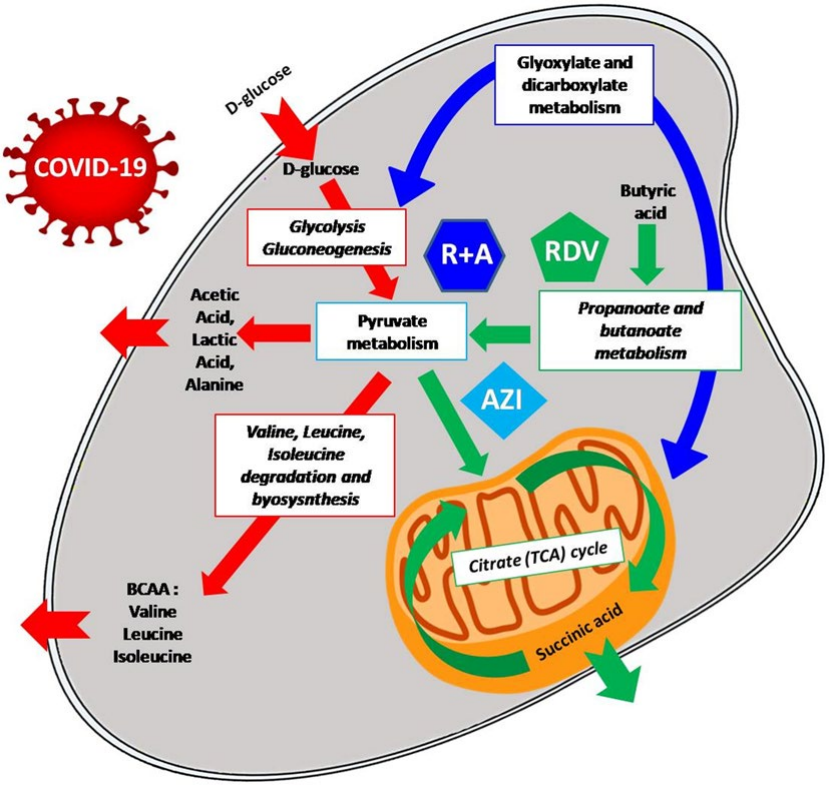
Conceptual graphical model of the main metabolic changes occurring in the host cell (VE6) in response to the examined treatments. Arrows underline the pathways that are most affected by SARS-CoV-2 infection (red) and explain the metabolic reprogramming induced by the drug pre-treatment either using RDV alone (green), AZI alone (cyan), or combined R + A (blue).

The primary reported metabolic changes and reprogramming in VE6 cells may be tracked by following the scheme illustrated in Fig. 5. With the aid of a thorough comparison with earlier in-vitro and in-vivo research, we examine these observations independently in the following sections in light of our NMR-based metabolic footprint evidence.

### Effect of SARS-CoV-2 infection on VE6 exometabolomes

The significant uptake of glucose by the cells from the extracellular medium, and the consequent conversion of glucose through glycolysis, are well-characterized effects of SARS-CoV-2 infection^17^. Glycolysis is a key metabolic process that sustains cell life, but among the recognized changes brought on by SARS-CoV-2, the glycolytic pathway is known to undergo a considerable transformation, usually as a result of mitochondrial oxidative damage^18–20^. Confirmation of heightened intracellular reactive oxygen species (ROS) levels was additionally substantiated through indirect assessments involving lipid oxidation products within cellular lysates. Specifically, this was accomplished by scrutinizing ^1^H NMR spectra of cellular lysates and juxtaposing spectral fingerprints associated with hydroperoxides (refer to Figure S4) between infected cells and those subjected to treatment (either with RDV monotherapy or the RDV and AZI combination). Notably, our analysis revealed a discernible reduction in hydroperoxide species within cells treated with RDV alone or the drug combination compared to untreated infected cells, thereby corroborating the efficacy of the administered treatments in mitigating oxidative stress.

Adenosine triphosphate (ATP), which is the energy source for the cell when it is supplied with oxygen, as well as glycolysis when oxygen is not present, are produced by mitochondria.

Following SARS-COV-2 infection, e.g. in patients with severe COVID-19, cells exhibit hyperglycolysis, which is characterized by the preferential synthesis of ATP through the glycolytic route as well as the suppression of oxidative phosphorylation^21^. This is caused by a metabolic reprogramming of the host glycolysis^20^, where pyruvate dehydrogenase (PDH) activity is lowered while lactate dehydrogenase (LDH) levels are elevated. Based on prior evidence, the production of lactate, accompanied by the activation of LDH and inhibition of pyruvate dehydrogenase (PDH), appears to promote the viral infectious milieu^17,20,21^. This enzymatic reconfiguration results in the suppression of oxidative phosphorylation and the onset of aerobic glycolysis or hyperglycolysis^20,21^. Critically ill COVID-19 patients had higher levels of various energy-related metabolites, such as pyruvate and lactate^22–24^. Therefore, it has been suggested that circulating pyruvate levels can be used as a predictor of critical COVID-19^24^.

According to our MSEA results, as shown by the significant effects on the pyruvate metabolism and the glycolysis/gluconeogenesis pathway, SARS-CoV-2 infection appears to effectively force VE6 cells to have increased glucose concentrations from extra-cellular medium and altered energetic metabolism. This is likely related to mitochondrial oxidative damage, upregulation of intracellular ROS generation, and increase of cellular injury and stress^25,26^. A considerable amount of lactate is also secreted as a consequence of the infection's overall effects. Similarly, high circulating LDH is frequently observed in human subjects affected by severe SARS-CoV-2 disease, as a consequence of derived sepsis shock^22,23^. However, lactic acidosis in the peripheral blood of patients with severe COVID-19 is rarely reported by studies, even when LDH levels are elevated, and serum lactate levels are typically not related to critically ill COVID-19 patients^22^. Several hypotheses have been proposed to elucidate this phenomenon, including the rapid utilization of lactate by the originating cells or its clearance by the liver and kidneys through the Cori cycle, consequently enhancing glucose synthesis. Our observations, alongside findings from complementary studies, lend greater support to the latter hypothesis due to its alignment with our results and those reported in the literature^18,19^.

The amino acid profiles of the VE6 exometabolomes are also altered by virus-induced metabolic changes, with an increase in branched-chain amino acids (BCAAs) such as leucine, valine, and isoleucine, as well as aromatic like tyrosine and histidine. Previous studies have addressed the importance of determining the amino acid profile in terms of both diagnosis and supplementation in SARS-CoV-2-affected patients^27–29^. In particular, an increased BCAAs level has been observed in SARS-CoV-2-affected patients, as a result of enhanced protein breakdown caused by related hypermetabolic status^28,29^. In such pathological situations, BCAAs become a very important energy resource for the muscles and reflect increased ROS generation and inflammation, thus being responsible for triggering the immune response^30^.

The immune-regulatory and pro-inflammatory lipid mediators were also found to be perturbed in a number of ways in SARS-CoV-2-infected human participants, according to recent hints of evidence^31,32^. When viruses invade host cells, they indeed release cytokines and interferon, causing inflammation and affecting viral survival and reproduction^33^.

These changes are mostly the result of the adipose tissue being lipolyzed, which causes triglyceride build-up in the plasma^34^. This phenomenon was previously associated with the presence of an excess of acetyl-CoA, due to the reduction of its oxidation metabolism^35^. As a result, the majority of acetyl-CoA metabolism is switched to the synthesis of ketone bodies, resulting in elevated levels of butyric and acetic acids in the blood^35^. The results of our MSEA, which show that the Pantothenate and CoA Biosynthesis route is one of the most affected by viral infection in VE6 cells, supported the previously described experimental evidence.

Furthermore, there have been reports of a down-regulation in the glyoxylate and dicarboxylate metabolism pathway, one of the most enriched pathways according to our MSEA, following SARS-CoV-2 infection. Glyoxylate and dicarboxylate metabolism includes a series of enzymatic reactions involving carbohydrates and fatty acids or two-carbon precursors that are known to play vital roles in cell energy supply under stress^36,37^. The disruption of this metabolic pathway is plausibly associated with the severity of COVID-19, as it has been observed to be down-regulated in severe cases compared to those with mild-to-moderate SARS-CoV-2 infection^38^.

### Effect of Remdesivir on VE6 infected cells

According to our MSEA analysis, when RDV is administered to SARS-CoV-2 infected VE6 cells, an extensive metabolic shift toward the TCA cycle is observed, also evidenced by a massive release of one of its major end-products (succinic acid) in the extracellular medium.

Also notably, the levels of several secreted metabolites appeared down-regulated in response to RDV administration when compared to untreated (both the infected and non-infected) VE6 cell lines. In particular, after RDV administration, the glucose uptake in infected VE6 cells appears to be almost in line with the non-infected VE6 cell line.

Moreover, according to our MSEA results, the metabolic impact of the glycolytic-gluconeogenetic pathway appears reduced in comparison with both the infected and non-infected VE6 cells. Previous studies confirm our findings about RDV-induced alteration of cell glycolysis and energy metabolisms: for example, Merches et al.^39^ recently observed an increased lactate secretion in an RDV concentration-dependent manner in H9c2 cells. Additionally, similarly to our findings, Du et al.^40^ observed a significant down-regulation of several rat blood metabolites after RDV intravenous treatment, including amino acids, choline-derivates, and nucleotides.

The observed increased consumption of short-chain fatty acids (SCFAs) appears to be one of the alternative possible pathways explaining the reported enhanced activity of the TCA cycle in RDV-treated VE6 cells. In our study, SCFAs are mostly represented by butyric acid, as denoted by its relevant decrease in the extra-cellular medium. This observation is further supported by the relevant enrichment of the propanoate and butanoate metabolisms in MSEA. In mammals, SCFAs are crucial substrates for both energy metabolism and anabolic activities^41^. In-vivo, SCFAs are mainly formed in the liver, through the peroxisomal β-oxidation of long-chain fatty acids (LCFAs)^42^. As was previously noted^43^, SCFAs tend to accumulate in tissues under pathological circumstances, impairing the function of the mitochondrial respiratory complexes^44^. When human macrophages were treated with SARS-CoV-2 free proteins^45^ several phenotypic and metabolic changes were observed. In particular, the same authors discovered that several metabolic pathways, including the Warburg effect, vitamin K, and propanoate, were enhanced. In order to ascertain the effects of RDV on mitochondrial respiration, or the disruption of cellular energy, Fišar and Hroudová^46^ measured the in-vitro effects of RDV, in particular the effects of RDV on the overall function of the respiratory chain (complex I-, II-, and IV-linked respiration), in isolated mitochondria. It was found that RDV caused small or negligible activity reduction of the respiratory chain complex I and II, at drug concentrations below 10 μmol/L.

Clinical research showed that RDV therapy increased the liver's alanine aminotransferase (ALT) enzyme activity in both healthy patients and those with other viral diseases^47^, thus supporting the observed perturbation of alanine, aspartate, and glutamate metabolism. The same studies also discussed the potential adverse effects of used RDV dosages. However, there is currently a lack of information and no particular investigations on the potential dosage-related toxicity of RDV in-vivo. In the present investigation, the used RDV dose was remarkably lower (2 μM) than the estimated in vitro toxicity threshold concentration (~ 10 μM)^48^. Moreover, previously documented cytotoxicity assays carried out in the same in-vitro model used for our NMR analysis, have displayed no detrimental effects in the examined cells at this concentration^49^.

Although our NMR-based metabolomics analyses unveil significant metabolic alterations in SARS-CoV-2-infected VE6 cell lines upon exposure to the antiviral agent remdesivir (RDV), leading to pronounced perturbations in the cell exometabolome, conversely, RDV treatment of uninfected cells did not elicit substantial changes in the exometabolome profile. Notably, principal component analysis (PCA) of uninfected VE6 cell exometabolomes exhibited distinct clustering patterns, with clear differentiation observed between cells treated with azithromycin (AZI) either alone (VE6-AZI) or in combination with RDV (VE6-R + A), relative to untreated controls (VE6-). However, clusters corresponding to RDV-treated uninfected cells (VE6-RDV) demonstrated considerable overlap with untreated counterparts, contrasting the distinct metabolic effects of RDV observed in infected cell lines, where RDV treatment alone highlighted a pronounced metabolic response. These findings strongly suggest that the metabolic impact of RDV is primarily confined to its interaction with the virus, exerting minimal influence on the metabolisms of VE6 cells in the absence of viral particles.

### Metabolic effect induced by AZI alone and RDV- AZI drug cocktail

The rationale behind selecting azithromycin (AZI) for COVID-19 treatment in our study stems from a broader investigation into repurposed drugs for COVID-19 therapy, as outlined in a previous publication^49^. This prior work emphasized the challenges encountered in finding effective antiviral treatments for hospitalized COVID-19 patients and proposed the exploration of synergistic drug combinations to overcome these obstacles. Through comprehensive screening of over 100 potential antiviral drugs, including AZI, in a Vero E6 cell culture model, the authors identified promising candidate drugs with documented antiviral activity. Subsequent testing revealed that combinations of RDV with either AZI or ivermectin exhibited synergistic inhibition of SARS-CoV-2 replication without inducing antagonistic effects. Notably, the RDV-AZI combination demonstrated enhanced potency, significantly lowering the concentration required for complete virus inhibition compared to monotherapy. Furthermore, it was found that AZI antiviral activity was augmented when combined with RDV, with implications for achieving effective lung concentrations in vivo, a critical consideration for treating respiratory viruses such as SARS-CoV-2. While monotherapy with AZI typically fails to reach the necessary lung concentrations for complete virus inhibition, combination therapy with RDV enables the attainment of therapeutic levels in the lungs for an extended duration. Building upon these findings, our current study aims to elucidate the molecular metabolic effects of these selected drug combinations on the same cellular model, thereby enhancing our understanding of their therapeutic mechanisms and potential implications for COVID-19 treatment outcomes.

Clinically, AZI is routinely utilized to modulate immune responses in patients with chronic inflammatory lung diseases^50^ and this benefit appears to be most applicable to subsets of patients who are older and specifically affected by respiratory diseases^51,52^. The effectiveness of such drug therapies is thought to mainly be due to the ability of AZI to reduce pro-inflammatory cytokine production and decrease neutrophil influx^53,54^. Both in-vitro and in-vivo studies show that AZI administration reduces cellular oxidative stress^55,56^, for example that induced by diabetes^57^.

We discovered that, despite having different p-values, the most enriched pathways for the metabolic impacts of AZI closely resemble those discovered for the pair VE6^+^ versus VE6^−^, namely glycolysis/gluconeogenesis, pyruvate, and glyoxalate and dicarboxylate metabolisms. Other metabolisms we found significantly enriched by AZI treatment are those related to alanine, aspartate, and glutamine and again, although to a lesser extent if compared with RDV treatment, the TCA cycle.

Previous research on using AZI to treat pneumococci showed that it had the least adverse effects on metabolism when compared to other studied antibiotics (cefotaxime, teixobactin-Arg10, and moxifloxacin)^58^. In the same study, glycolysis appears to be scarcely affected by AZI, demonstrating that the antibiotic treatment did not affect the energy balance of the bacteria. Moreover, except for the metabolism of glutamine/glutamate, no change in amino acid metabolisms, and no significant uptake of amino acids was also discovered. This would support the theory that the viral effect is the main factor influencing the metabolic fate of infected cells and that AZI monotherapy primarily had a modulatory influence on the most important cellular metabolisms in the current investigation (as shown by the p-value of our MSEA). However, given the importance of the TCA cycle in our MSEA and taking into account the observed decrease of SCFAs in the extra-cellular medium, as previously discussed for RDV treatment, we would be inclined to believe that AZI could have some effects on cellular energy metabolism, supporting previous findings that the drug causes mitochondrial toxicity, upregulation of glycolysis-related genes, and the induction of aerobic glycolysis^58,59^.

Further substantiating the modulatory effect of AZI on critical cellular metabolic pathways in VE6 cells, our NMR-based MVDA elucidated significant metabolic perturbations induced by AZI administration, whether administered independently (VE6-AZI) or in combination with Remdesivir (RDV) (VE6-R + A) in both uninfected and infected cellular models. This observation indicates a significant impact of AZI on the fundamental metabolic processes of VE6 cells even in the absence of viral presence, particularly when compared to the sole administration of RDV.

Overall, these pieces of evidence suggest that RDV and AZI, either alone or in combination, can reprogram the glycolic-gluconeogenesis pathways in the host cell to impair the mitochondrial oxidative damage occurring in the primary phase of SARS-CoV-2 virus interaction. Interestingly, the metabolic effects induced by the combined administration of RDV and AZI in SARS-CoV-2-infected VE6 cells result quite close to those induced by AZI monotherapy, apart from a less evident metabolic shifting towards the TCA cycle in the cells treated with the drug cocktail, which seems to be indicative of the influence of RDV-treatment.

We notice that the combination of AZI and RDV determines a downregulation of numerous metabolites in the extracellular media, albeit to a little lesser extent than RDV treatment alone, when the specific metabolites implicated in the aforementioned pathways are taken into account. In summary, the closeness of cellular metabolic conditions following AZI therapy alone and in conjunction with RDV is corroborated by the study of the most significantly changed metabolites found in the extracellular media of VE6 cells.

In one recent study, De Forni et al. have shown major beneficial effects measuring the antiviral activity using the identical dosage combination of RDV and AZI on the same in vitro model compared with the use of single drugs^49^. Moreover, earlier clinical research has also suggested that the therapeutical effect of RDV in COVID-19 pneumonia may be potentiated by AZI administration^60,61^. Although we presented here a complete analysis of the most relevant metabolic features of AZI + RDV co-treatment in-vitro, further investigations are certainly required to more clearly ascertain the molecular mechanism of action, effect on cellular metabolism efficacy, and safety of the synergy of these two candidate drugs.

## Conclusions

As a general proof-of-concept, our ^1^H NMR metabolic footprint analysis demonstrates to be a powerful tool to investigate the metabolic challenges occurring in VE6 exometabolomes subjected to SARS-CoV-2 infection and administration of RDV and AZI as preventive treatment as a single or combined therapy.

Our findings highlight the important role played by several metabolic pathways involved in cellular metabolic homeostasis and targeted by the SARS-CoV-2 virus during its initial interaction with the host, even though they should not be regarded as conclusive given the top-down nature of metabolomics approaches. Our data also give further experimental support to the molecular actions of single and combined administrations of two promising repurposed drugs (RDV and AZI) to reprogram host cell metabolism to impair SARS-CoV-2 infection and replication.

Our NMR metabolic footprinting analysis also emphasizes the need for deeper understanding of the host metabolic reprogramming induced by SARS-CoV-2 infection and preventive drug therapies to produce meaningful references for the successful application of precision therapy in SARS-CoV-2 affected patients.

## Material and methods

### Cell culture and NMR sample preparation

Details on chemicals used are reported in Supplementary Note S1.1. Vero E6 (VE6) cells were cultured and treated with the two tested drugs (RDV and AZI) as described in the previously published paper^49^. Briefly, Vero E6 cells (VE6) (*Cercopithecus aethiops*, kidney, ATCC CRL-1586) were initially seeded at a density of 500,000 cells/well into a 96-well plate in 3 ml Dulbecco’s Modified Eagle Medium (DMEM) supplemented with 1% glutamine, 1% penicillin/streptomycin and 10% fetal bovine serum (FBS) at 37 °C and 5% CO_2_. Approximately 1 h after seeding, VE6 cell supernatants (except the untreated infected and uninfected cell lines) were replaced with 2% of complete medium (CM) containing 1 μM of remdesivir and/or 10 μM of azithromycin drugs (i.e. either alone or in combination). Then, within one hour, all cell lines were infected with selected SARS-CoV-2 strains, and additionally cultured for a further 72 h. At the end, 1 ml of supernatant was collected from each plate, rapidly deep frozen in N_2,_ and stored at -80 °C until the next processing steps. Cell lysates were similarly collected from each plate 72 h after infection, rapidly deep frozen, and stored at -80 °C until NMR analysis of the lipid extracts. Lipids from cell lysates were extracted by vortexing the lyophilized sample in 1 ml of deuterated chloroform for 1 min after equilibrating the sample temperature in ice, using amber glass vials to avoid direct light. and pooling samples from 5 plates to generate one NMR sample per treatment.

VE6 cell supernatants used for NMR footprint analysis were prepared in agreement with the procedure detailed in Supplementary Note S1.2 and based on literature guidelines^62^. In total, twenty replicates of VE6 cells supernatants, coming from the following treatments, were prepared for ^1^H NMR-based metabolomics inspection: (i), untreated and uninfected cells (VE6^−^); (ii), untreated and SARS-CoV-2 infected (VE6^+^) cells; (iii), uninfected cells treated with 2 mM of RDV (VE6-RDV); (iv) SARS-CoV-2 infected cells and treated with 2 μM of RDV (VE6^+^RDV); (v), uninfected cells treated with 10 mM of AZI (VE6-AZI);

(vi) SARS-CoV-2 infected cells and treated with 10 μM of AZI (VE6^+^AZI); (vii) uninfected (VE6-R + A) and (viii), SARS-CoV-2 infected cells (VE6^+^R + A) treated with a combination of the same doses of RDV and AZI. The cell culture service was outsourced to ViroStatics S.r.l. and carried out in accordance with Porto Conte Ricerche’s procedures in BSL3 facilities controlled by Porto Conte Ricerche (Alghero, Sassari, Italy).

### NMR acquisition

All ^1^H NMR measurements were performed at room temperature (292.0 ± 1.0 K) using a Bruker Avance 600 MHz spectrometer (Bruker Biospin, Karlsruhe, Germany), equipped with a 5 mm BBI probe. One-dimensional (1D) ^1^H NMR spectra were recorded through a simple pulse-acquisition-relaxation delay sequence with suppression of residual water signal during the relaxation delay (Bruker sequence *zgpr*) and processed using the software Bruker TOPSPIN (version 2.1).

The NMR acquisition parameters of supernatants were the following: number of scans (NS) = 64; spectral width (SW) = 7002.8 Hz; size of FID (TD) = 65,536; relaxation delay (D1) = 27 s; acquisition time (AQ) = 3.0 s. ^1^H NMR identification (assignment) of metabolites in VE6 cells supernatants was made by acquiring two-dimensional (2D) homonuclear (^1^H–^1^H total correlation spectroscopy, TOCSY) and heteronuclear (^1^H–^13^C heteronuclear single quantum coherence, HSQC) spectra. ^1^H NMR assignment was further confirmed by comparing the chemical shifts with the reference spectra of pure compounds available in online databases, including the Human Metabolome^63^ and the Biological Magnetic Resonance Data Bank (BMRB)^64^.

The NMR acquisition parameters of cell lysate lipids were the following: number of scans (NS) = 256; spectral width (SW) = 7002.8 Hz; size of FID (TD) = 32 k; relaxation delay (D1) = 3 s; acquisition time (AQ) = 2.34 s.

### NMR data pre-processing

1D NMR spectra were pre-processed using a NMRProcFlow tool (version 1.4^65^)^66^ as detailed in Supplementary Note S1.3. Twenty-two NMR-identified metabolites with their relative quantities (integral values reported in arbitrary units, a.u.) were selected for subsequent statistical data analysis, according to previous recommendations^67^. Representative 1D ^1^H NMR profiles, tabular average mean, and standard deviations of selected NMR data (integrals, a.u) are freely available at Zenodo (https://zenodo.org/records/10158609).

### Statistical data analysis

NMR data were imported into the Statistical Analysis module included in the web-based software MetaboAnalyst version 5.0^68^ for multivariate and univariate data analysis^69^. For multivariate purposes, ^1^H NMR data were preliminary constant sum normalized, logarithm-transformed and Pareto scaled. Unsupervised principal component analysis (PCA) was initially carried out to identify outlier samples and possibly observe group clustering. Additionally, the related PCA scores dendrogram was also generated, by using the PCA2Tree tool, to provide a quantitative measure of the significance of similarity/difference between the observed clusters. PCA2Tree computes the dendrograms using Mahalanobis distances and reports p-values for the null hypothesis at all internal branches, where p < 0.05 indicates a statistically significant difference^70^. A more robust supervised multivariate approach, employing partial least square discriminant analysis (PLS-DA), was also carried out to better characterize sample grouping (1. infected vs. uninfected and 2. infected and treated vs. infected VE6 cells exometabolomes). For each PLS-DA model, cross-validation (CV) was also performed, by using the parameter Q2 as indicative of the predictive ability of the model. Good predictions will have high Q^2^ computed values^71^.

To get proper information on the metabolites responsible for observed sample clustering, the importance feature analysis based on univariate statistical discrimination was performed. In particular, no-parametric univariate ANOVA with Fischer’s LDS post-hoc test (with no preliminary data pre-processing) was carried out on NMR data using the same MetaboAnalyst’s Statistical Analysis module. p-values (p < 0.05) and associated false discover rate (FDR < 0.05) were adopted as significance threshold levels within all group inter-comparisons, which can be found in Zenodo (https://zenodo.org/records/10158609).

### Metabolite set enrichment analysis

Aiming to analyze each treatment-related metabolic perturbation, a Metabolite Set Enrichment Analysis (MSEA) was carried out using the MetaboAnalyst Enrichment Analysis module. For this purpose, the same NMR data preliminarily inspected by univariate and multivariate statistical analyses were used as input entries. Functional metabolic pathways were enriched utilizing the Kyoto Encyclopedia of Genes and Genomes (KEGG) database^72–75^, setting *Homo sapiens* as the model organism. Statically perturbed pathways were ranked in order of importance by considering both the pathway impact value computed from pathway topological analysis (indicated by Enrichment Ratio, ER) and the log of the p-value (indicated by the p-value). MSEA output data in tabular formats are freely available at Zenodo (https://zenodo.org/records/10158609).

### Supplementary Information

Supplementary Information.

## Data Availability

The datasets generated during and/or analyzed during the current study are available in the Zenodo repository, [https://zenodo.org/records/10158609]. Further data generated or analyzed are either included in this published article (and its Supplementary Information files) or available from the corresponding author on reasonable request.
