# Supplementary material for: The metabolic footprint of Vero E6 cells highlights the key metabolic routes associated with SARS-CoV-2 infection and response to drug combinations
Source: Sci Rep. 2024 Apr 4;14:7950. doi: 10.1038/s41598-024-57726-3 (PMC10995198; doi:10.1038/s41598-024-57726-3)
Supplement: Supplementary file 1 — Supplementary Information. [file 41598_2024_57726_MOESM1_ESM.docx]

Supplementary Information

**Metabolic footprint of Vero E6 cells highlights the key metabolic routes associated with SARS-CoV-2 infection and response to drug combinations**

Riccardo Melis^1^, Angela Braca^1^, Daniela Pagnozzi^1^ and Roberto Anedda^1^*

^1^ Porto Conte Ricerche s.r.l., S.P. 55 Porto Conte-Capo Caccia, Km 8.400 Loc. Tramariglio, Alghero (SS), Italy

* Corresponding Author: anedda@portocontericerche.it

Supplementary Tables

**Table S1**. **^1^**H NMR assignments of the main metabolites identified in the secreted medium of VE6 cells. The signals selected for relative quantifications (buckets) according to Supplementary Note S1.4 are highlighted in bold.

|  |  | **^1^H(ppm) and multiplicity** |
| --- | --- | --- |
| **Number** | **Compound** |  |
| 1 | **Lipids** | **0.87** (m, broad) |
| 2 | **Butyric acid** | **0.89 (t)**, 1.54 (m), 2.11 (t) |
| 3 | **Isoleucine** | **0.94(t)**, 1.01(d), 3.66 (d) |
| 4 | **Leucine** | **0.95(d)**, 0.96(d), 3.70 (d) |
| 5 | **Valine** | 0.99(d)，**1.03(d)**，3.59 (d) |
| 6,7 | Unknown | 1.10 (d) , 1.12 (d), 1.10 (d) , 1.23 (d), 1.45(d) |
| 8 | Propionic + Butanoic acid | 1.17 (t), 2.16 (q) |
| 9 | **Lactic acid** | **1.33(d)**，4.11(q) |
| 10 | **Alanine** | **1.48(d)**, 3.78(q) |
| 11 | Lysine + Arginine | 1.72(m), 1.89 (d., ov.), 3.02 (t) |
| 12 | **Acetic Acid** | 1.91(s) |
| 13 | **Proline** | **1.99(m)**, 2.22 (m) |
| 14 | **Methionine** | **2.14 (m)**, 2.63(t) |
| 15 | **Glutamic Acid** | 2.10(m), **2.34(m)** |
| 16 | **Glutamine** | 2.12(m), **2.43(m)** |
| 17 | **Pyruvic acid** | **2.37(s)** |
| 18 | **Succinic acid** | **2.41(s)** |
| 19 | Citric + Malic acid | 2.53(d)，2.69(d) |
| 20 | Creatine+Phosphocreatine | 2.87(s), 3.93(s) |
| 21 | **Choline** | **3.20(s)**，3.66(m) |
|  |  |  |
| 22 | **D-Glucose** | 3.24(dd)，3.46(m)，3.76(dd)，**4.66 (d)**, **5.25 (d)** |
| 23 | **Tyrosine** | 6.90(d)，**7.20 (d)**，7.33(d) |
| 24 | **Histidine** | **7.09(s)** |
| 25 | **Phenylalanine** | 7.33 (d)**，7.38 (t)** |
| 26 | **Tryptophan** | **7.56(d)**，7.62(d) |
| 27 | **Adenosine diphosphate (ADP)** | **7.89(s)** |
| 28 | **Formic acid** | **8.36(s)** |
| 29,30 | Unknown | 8.76(d), 8.78(d), 8.89 (s) |

Supplementary Figures


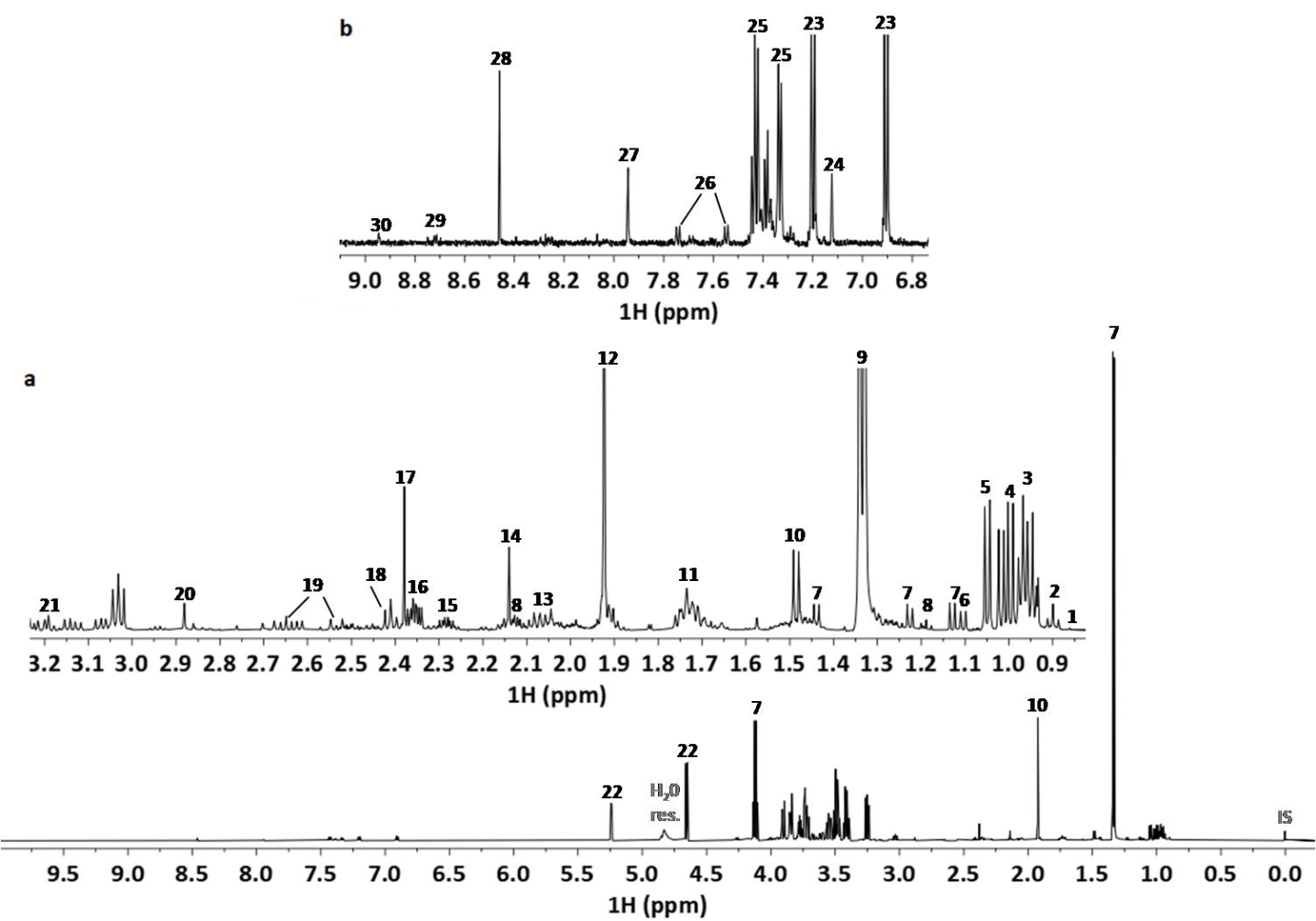


**Figure S1.** Representative 1D ^1^H NMR spectra (600.13 MHz) showing signal assignments for the main metabolites identified in the secreted medium of non infected and untreated Vero E6 (VE6^+^) cell. Metabolites annotated are in agreement with previous assignments reported in **Table S1.** Additional notes**:** IS = NMR internal Standard (TMSP-d4); H_2_O res. = water residue in D_2_O.

**Figure S2.** 2D Score plot from supervised MVDA (PLS-DA) analysis related to the comparison between : a) VE6^+^ vs. VE6^-^ ; b) VE6^+^RDV vs. VE6^+^; c) VE6^+^AZI vs. VE6^+^; and d) VE6^+^ R+A vs. VE6^+^ cells.

**Figure S3.** Cross validation test of PLSDA analysis. (a): Cross validation test of data of **Figure S2a**; (b): Cross validation test of data of **Figure S2b**; (c): Cross validation test of data of **Figure S2c**; (d): Cross validation test of data of **Figure S2d.**

**Figure S4.** Box-plots showing all relative metabolite levels (NMR integrals, a.u.) quantified in the examined VE6 esometabolomes: VE6^-^ (black boxes); VE6^+^ (red boxes); VE6^+^RDV (green boxes); VE6^+^AZI (cyan boxes); VE6^+^R+A (blue boxes).


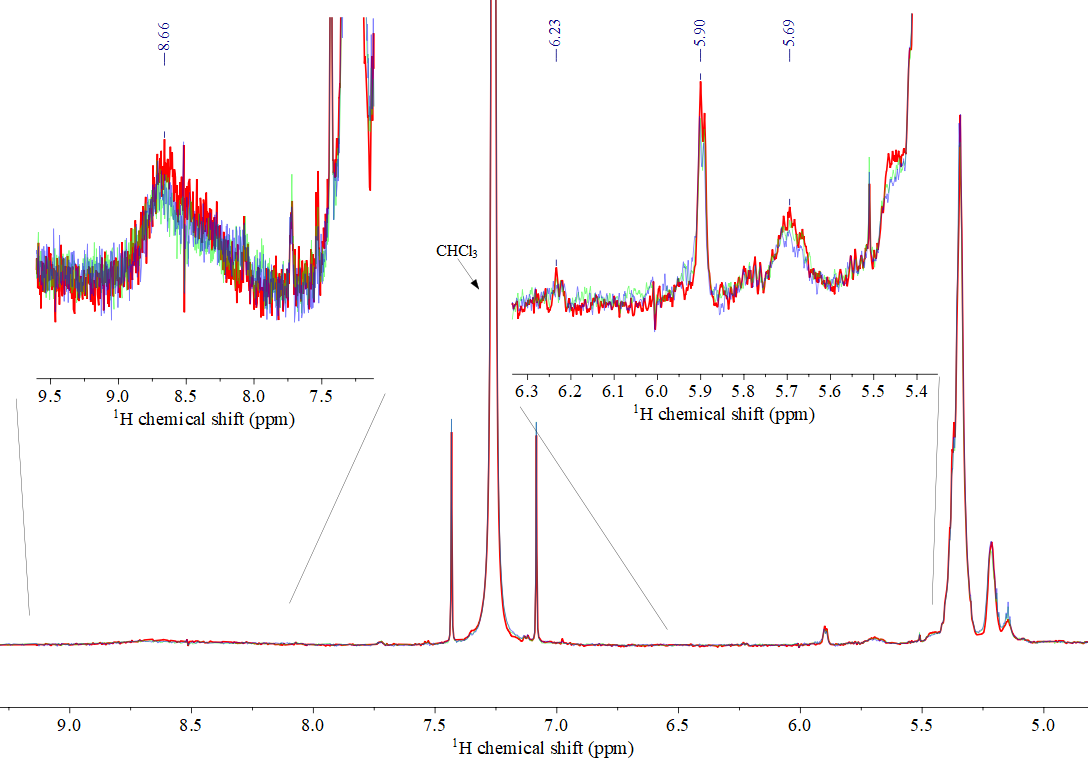


**Figure S5.** Proton NMR spectra of the lipid portion of SARS-CoV-2-infected (VE6+) cell lysates (red) and cell lysates following drug pre-treatment with RDV alone (green) or RDV+A (cyan). The signals at 5.44 ppm, 5.69 ppm, 5.90 ppm, 6.23 ppm, and 8.66 ppm indicate the presence of hydroperoxy groups in primary oxidation products of fatty acids. Spectral expansions are reported as onset figures,which refer to the regions indicated by grey lines. The intensity of these signals is notably higher in the extracts of VE6+ lysates compared to cells treated solely with RDV or the combination of drugs. This observation lends support to the hypothesis of elevated reactive oxygen species (ROS) production in untreated cells following infection.Inizio modulo

To support assignments please refer to Martin-Rubio, Ana S., Patricia Sopelana, María L. Ibargoitia, and María D. Guillén. 2021. "^1^H NMR Study of the In Vitro Digestion of Highly Oxidized Soybean Oil and the Effect of the Presence of Ovalbumin" Foods 10, no. 7: 1573. https://doi.org/10.3390/foods10071573 and references therein.

**Supplementary Note S1.1**

**Chemicals**

Potassium dihydrogen phosphate (KH_2_PO4) and phosphoric acid (H_3_PO4) were purchased from Sigma-Aldrich (Merck KGaA, Darmstadt, Germany). Phosphate buffered saline (PBS) in D_2_O used for NMR analysis was prepared by dissolving 0.37 gr of KH_2_PO4 and 0.27 gr of H_3_PO4 in 100 ml of D_2_O. Deuterium oxide (D_2_O, 99.9% D) and deuterated sodium trimethylsilylpropionate (TMSP-2,2,3,3-d4, 98.9% D) were purchased from Cambridge Isotope Laboratories Inc. (Andover, MA, USA).

**Supplementary Note S1.2**

**NMR sample preparation**

VE6 cell frozen supernatants were initially heated at 56 °C for 30 min using a dry block (ThermoMixer Comfort, Eppendorf SE, Hamburg, Germany) in order to inhibit the viral infectivity. Samples were then dried for 7 hour at 30 °C using a vacuum concentrator (Concentrator Plus, Eppendorf SE, Hamburg, Germany) to remove the major part of water. The obtained dried pellets were further freeze-dried (LIO2000P, 5Pascal, Milan, Italy) over-night and re-suspended in 900 μl of PBS 50 mM pH 6.8 in D_2_O, containing 0.1 mM TMSP as NMR chemical shift reference. Finally, samples were vortexed (14.000 g x 10 minutes at 4°C) and a fixed volume of 800 μl was transferred into 5 mm o.d. NMR tubes for subsequent analysis.

**Supplementary Note S1.3**

**NMR spectra pre-processing**

Raw 1D ^1^H NMR spectra (Bruker FID) were uploaded into NMRProcflow tool and automatically calibrated against the chemical shift of the internal standard, IS (TMSP-d4) at 0 ppm, full baseline corrected and aligned by means of hierarchical Cluster-Based Peak Alignment Algorithm (CLUPA). NMR signal areas were manually integrated by using variable-size bucketed function. Each integral area was then normalized with respect to the MSI and expressed as relative quantification in arbitrary unit (a.u.). To check the accuracy of NMR integration, average signal to noise ratio (S/N) was also computed for each integral: only unambiguous signals having the S/N higher than the lower limit of quantification (LLOQ) of S/N > 10 were taken into account for subsequent statistical analysis.
